# Crossed Renal Ectopia without Fusion—An Unusual Cause of Acute Abdominal Pain: A Case Report

**DOI:** 10.1155/2012/728531

**Published:** 2012-10-10

**Authors:** D. P. Ramaema, W. Moloantoa, Y. Parag

**Affiliations:** Diagnostic Radiology Department, Nelson R Mandela School of Medicine, University of Kwazulu-Natal, Private Bag 7, Congella, Durban 4013, South Africa

## Abstract

*Introduction*. Crossed renal ectopia is a congenital anomaly which usually goes unnoticed as most cases are asymptomatic. The majority, 90% of these are fused. *Case Presentation*. We report an unusual presentation of a case of crossed renal ectopia without fusion. Our patient is a 16-year-old adolescent male, previously fit and healthy, who presented with acute onset of abdominal pain. The clinical suspicion was that of an abdominal aortic aneurysm. Computed tomography with intravenous contrast revealed nonfused crossed renal ectopia. *Conclusion*. Although renal ectopia is an uncommon cause of acute abdominal pain, there should be an index of clinical suspicion in previously healthy individuals presenting with acute abdominal pain.

## 1. Introduction

Renal ectopia as the cause of abdominal pain is a diagnosis of exclusion after other possible causes have been excluded. Most cases remain undiagnosed because they remain asymptomatic [1]. From 20 to 30% of cases are incidentally diagnosed [2]. 

## 2. Case Presentation

**Figure 1:**
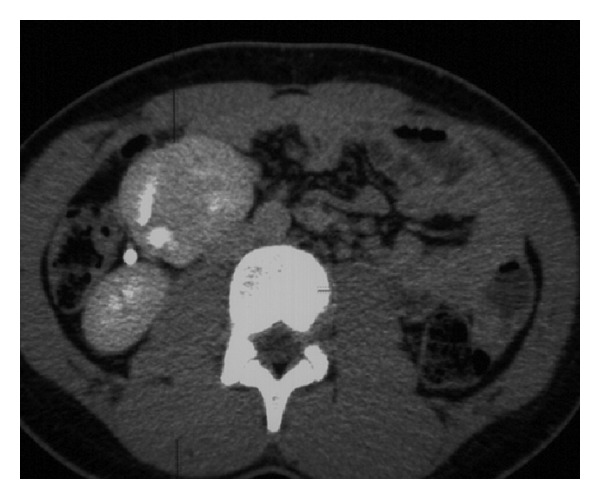
16-year-old adolescent male with left-to-right crossed renal ectopia. Axial CT scan with intravenous contrast-delayed phase demonstrates two normal enhancing kidneys on the right.

**Figure 2:**
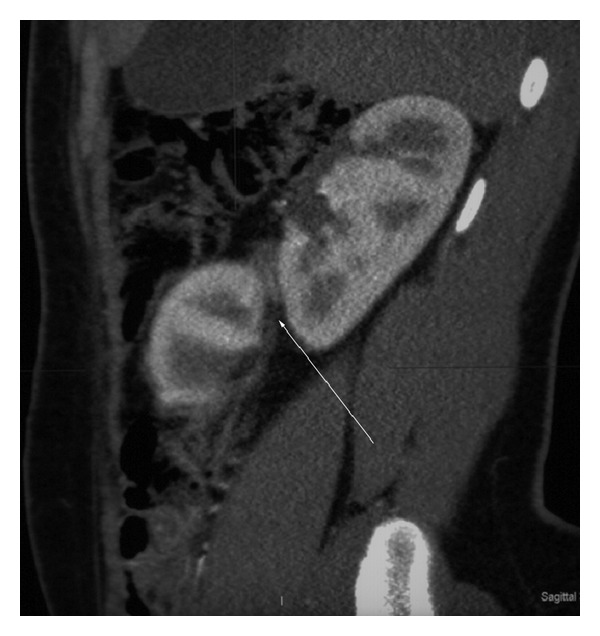
16-year-old adolescent male with left-to-right crossed renal ectopia. Sagittal CT scan with intravenous contrast, corticomedullary phase. Showing clear plane of separation between the two kidneys (white arrow), each kidney having its own Gerota's fascia.

**Figure 3:**
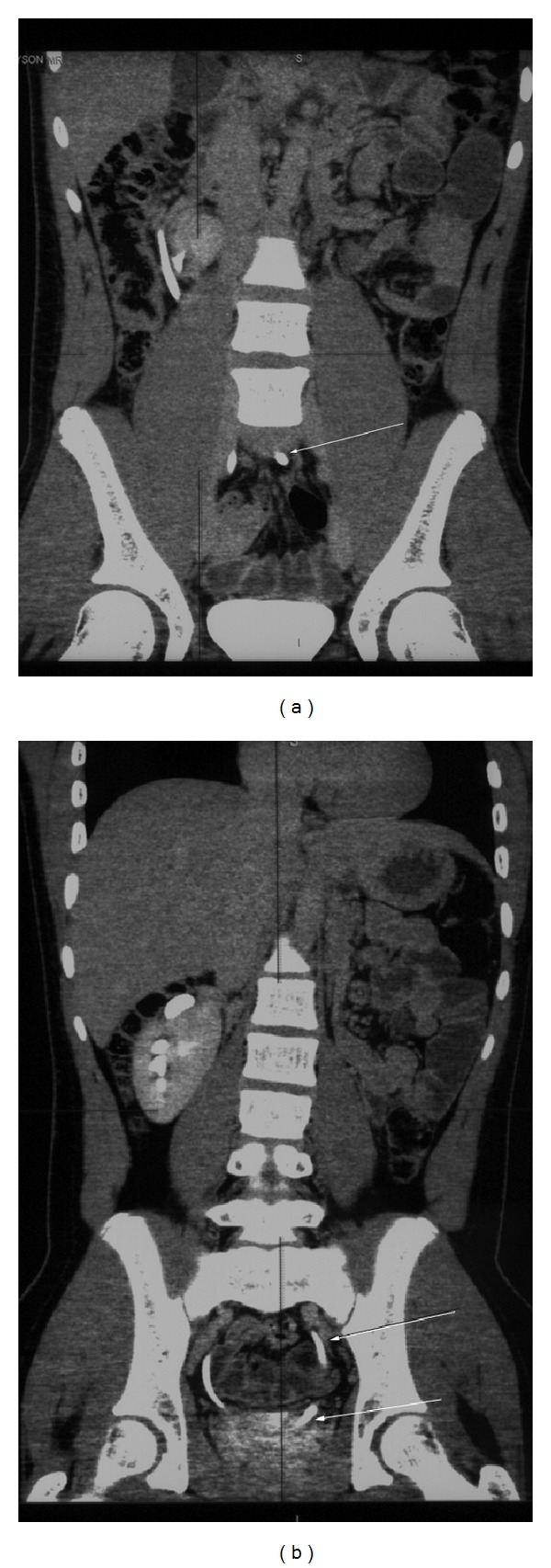
16-year-old adolescent male with left-to-right crossed renal ectopia. Coronal CT scan with intravenous contrast-delayed phase demonstrates the left ectopic ureter crossing midline (white arrow in (a)), to insert onto left side bladder (white arrows in (b)).

A 16-year-old adolescent male presented with acute abdominal pain of unknown cause. This was the first time the patient presents with abdominal pain. Based on the clinical examination, a diagnosis of abdominal aortic aneurysm was suspected. The patient had no background medical history. Initial blood investigations were normal. Serum urea and creatinine were normal. Chest X-ray and abdominal X-rays were normal. Computed tomography (CT) scan was requested to evaluate for the cause. CT scan revealed crossed ectopic left kidney without fusion to the right kidney. Both kidneys showed normal renal parenchymal enhancement (Figure 1). The left ectopic kidney was situated inferior to the right kidney. There was a clear plane of separation between the two kidneys, with each kidney having its own Gerota's fascia (Figure 2). There was rotation of the ectopic kidney, and the renal pelvis was anteriorly orientated. The ureter from the left ectopic kidney was seen crossing the midline to insert on the left side of the bladder (Figures 3(a) and 3(b)). 

Separate conventional intravenous urography (IVU) was not performed because the diagnosis was conclusive on CT scan.

## 3. Discussion

Most cases of crossed renal ectopia are asymptomatic and are noted incidentally during autopsy, screening tests, or during investigation for unrelated causes [1, 3]. Of these, the commonest form seen is the fusion variety, accounting for 90% of cases [4], having reported incidence of 1 in 7,500 autopsies. In contrast, the nonfused variety has been reported as 1 in 75,000 autopsies [3]. Four types of crossed renal ectopia have been described: type A, with fusion; type B, without fusion; type C, solitary crossed; type D, bilaterally crossed. Our patient had the uncommon type B variety. The presentation of acute abdominal pain was thought to be due to abdominal aortic aneurysm, even though there was no prior history. This is because renal ectopias are usually asymptomatic [1]. It was after exclusion of other pathologies that the cause of abdominal pain was attributed to renal ectopia. The occurrence of symptoms is seen more commonly in males, 2 : 1, and the left-to-right variety common, accounting for 51% of cases [5]. Our patient is also a male and demonstrated the left-to-right variety. However, the presentation of acute abdominal pain is very unusual, specifically with failure to demonstrate any associated complications or anomalies occurring at the same time. The renal function of both kidneys was normal, as evidenced by normal blood tests and normal renal cortical appearances after intravenous contrast administration. There was no hydronephrosis. The non contrast CT scan did not demonstrate any left renal calculi.

There are other tests which can be used to investigative renal ectopia. Radioisotope scans have been used [6]. Belekar demonstrated a nonfunctioning ectopic kidney by technicium-99m dimercaptosuccinic acid Tc99mDTPA scan [7]. G. Nursal used technicium-99m dimercaptosuccinic acid (Tc-99m DMSA) static and Tc-99m DTPA dynamic isotope studies in his two cases, for assessment of function and excretion [8]. Ultrasound can be used, but because our patient already had diagnostic CT scan, this was deemed unnecessary. Intravenous urography can be used to delineate renal excretion; however, delayed phase during CT scanning had been performed, where renal excretion was clearly demonstrated. If major surgery is planned, nephrotomography to define renal outlines and retrograde ureteroscopy with or without stenting are advised to define the collecting system and draining mapping [1]. 

Whereas our patient had no other explanation for the acute abdominal pain, all the other reported cases of crossed renal ectopia without fusion presenting with abdominal pain had other explanations. These include ipsilateral ureteral carcinoma [9], nonfunctioning kidney [7], and multicystic dysplasia [4]. Some causes of abdominal pain were from associated anomalies such as abdominal aortic aneurysm [10] and extrarenal pelvis [1]. This makes our case unique by virtue of exclusion of other causes of acute abdominal pain. We concluded that the sudden colic was attributed to a single episode of left urinary pathway obstruction associated with this nonfused crossed ectopia although there were no demonstrable complications. The patient was treated conservatively with analgesics and anti-inflammatory agents. He was discharged a day after the scan and remained well and asymptomatic on followup for a month after the acute episode.

## 4. Conclusions

Renal tract congenital anomalies should be suspected in previously asymptomatic patients who present with acute abdominal pain. Even though not common, the ectopic kidney varieties should be thought of. In our case, we conclude that a single temporary episode of left urinary pathway obstruction caused the severe colic.
